# Structured analysis, evaluation and report of the emergency response to a terrorist attack in Wuerzburg, Germany using a new template of standardised quality indicators

**DOI:** 10.1186/s13049-018-0555-5

**Published:** 2018-10-19

**Authors:** T Wurmb, N Schorscher, P Justice, S Dietz, R Schua, T Jarausch, U Kinstle, J Greiner, G Möldner, J Müller, M Kraus, S Simon, U Wagenhäuser, J Hemm, N Roewer, M Helm

**Affiliations:** 1 Subsection Emergency and Disaster Relief Medicine, Department of Anaesthesia and Critical Care, University Hospital of Wuerzburg, Oberdürrbacherstrasse 6, 97080 Würzburg, Germany; 2 Department of Anaesthesia and Critical Care, University Hospital of Wuerzburg, Wuerzburg, Germany; 3 Emergency Medical Services and firebrigade alerting for the counties of Kitzingen, Main-Spessart and the city of Wuerzburg, Wuerzburg, Germany; 4 Emergency Medical Service of the Bavarian Red Cross, Würzburg, Germany; 5 Medical Department, Government of Lower Franconia, Wuerzburg, Germany; 6 Emergency Medical Service of the Maltese Cross, Wuerzburg, Germany; 7 The Johanniter Rescue Emergency Services, Wuerzburg, Germany; 8 Fire and Rescue Integrated Control Centre Wuerzburg, Wuerzburg, Germany; 9 Medical Head of the Emergency Medical Services of Lower Fraconia, Wuerzburg, Germany; 10 Head of emergency pastoral care in the diocese of Würzburg, Wuerzburg, Germany; 11 Department of danger prevention and police operation by the police department of Lower Franconia (Chief Police Officer i.R.), Würzburg, Wuerzburg, Germany; 12 Department of Anaesthesiology and Intensive Care Medicine, Section Emergency Medicine, Federal Armed Forces Medical Hospital, Ulm, Germany

**Keywords:** Terror attack, Mass casualties, Evaluation, Quality indicators, Rescue mission

## Abstract

**Background:**

Until now there has been a reported lack of systematic reports and scientific evaluations of rescue missions during terror attacks. This however is urgently required in order to improve the performance of emergency medical services and to be able to compare different missions with each other. Aim of the presented work was to report the systematic evaluation and the lessons learned from the response to a terror attack that happened in Wuerzburg, Germany in 2016.

**Methods:**

A team of 14 experts developed a template of quality indicators and operational characteristics, which allow for the description, assessment and comparison of civil emergency rescue missions during mass killing incidents. The entire systematic evaluation process consisted of three main steps. The first step was the systematic data collection according to the quality indicators and operational characteristics. Second was the systematic stratification and assessment of the data. The last step was the prioritisation of the identified weaknesses and the definition of the lessons learned.

**Results:**

Five important “lessons learned” have been defined. First of all, a comprehensive concept for rescue missions during terror attacks is essential. Furthermore, the establishment of a defined high priority communication infrastructure between the different dispatch centres (“red phone”) is vital. The goal is to secure the continuity of information between a few well-defined individuals. Thirdly, the organization of the incident scene needs to be commonly decided and communicated between police, medical services and fire services during the mission. A successful mission tactic requires continuous flux of reports to the on-site command post. Therefore, a predefined and common communication infrastructure for all operational forces is a crucial point. Finally, all strategies need to be extensively trained before the real life scenario hits.

**Conclusion:**

According to a systematic evaluation, we defined the lessons learned from a terror attack in 2016. Further systematic reports and academic work surrounding life threatening rescue missions and mass killing incidents are needed in order to ultimately improve such mission outcomes. In the future, a close international collaboration might help to find the best database to report and evaluate major incidents but also mass killing events.

## Background

Systematic descriptions and academic evaluations of civil rescue operations during terror attacks or other life threatening mass casualty incidents are urgently needed. After the Paris terrorist attacks, some high-ranking articles, describing the events of the night of the 13th of November 2015, and the “lessons learned” from these attacks have been published [1–4].

Despite these very important publications, there have not been any systematic and scientific evaluations of the most recent terror attacks so far. Qualified experts however clearly state the need for such evaluations [5, 6]. Systematic reports will allow for the description, assessment and comparison of civil emergency rescue operations during terrorist attacks and they may serve as a very important basis in order to define and communicate the lessons learned [5–9]. For example the Utoja terror attack was reported systematically according to a template of indicators published by Fattah et al. [8].

Wuerzburg (Bavaria/Germany) experienced the first of a series of terrorist attacks and other mass killing events within Germany. A terrorist attacked train passengers with an axe and a knife on the eve of the 18th of July 2016. Four people were seriously injured before the train came to an emergency halt in the middle of a residential area. The emergency stop led to the offender halting the attack and escaping on foot. During his escape he injured a 5th person severely with an axe strike. Another person was injured when fleeing the train. The terrorist was later captured by a special police task force and shot in self-defence after attacking the police officers.

The evaluation of the rescue mission resulted in the realisation that a systematic description and academic evaluation of the operation was urgently needed. As a first step we developed a template of quality indicators and operational characteristics, which allow for the description, assessment, reporting and comparison of civil emergency rescue operations during terrorist attacks or shooting rampages [10]. We than started a systematic three step evaluation process using this template of quality indicators. The result is a systematic report and the definition of specific lessons learned from the terrorist attack in Wuerzburg.

Some models and templates for such systematic descriptions of rescue missions for mass casualty incidents have been described internationally [7–9]. One of them was presented by Fattah et al. [8]. Although these templates are very well designed, we decided to define a separate template, which focus on mass killing events and which would be better adapted to the German Emergency Medical Service’s (EMS) conditions [10]. For our understanding, it would not have been helpful to extend the existing templates as we wanted to learn more about the special conditions during mass killing events and we wanted to adapt these lessons learned directly to the German EMS System.

## Methods

Under the direction of the subsection “Emergency and Disaster Relief Medicine” of the Department of Anaesthesia and Intensive Care at the University Hospital Wuerzburg, a panel of experts was established in order to perform the evaluation process. The expert group consisted of leading representatives of the Emergency Medical Services of Wuerzburg, the offices for Emergency Medical Services and for Fire Brigade Alerting, the government of Lower Franconia, the emergency pastoral care, the police forces and the head of the subsection emergency and disaster relief medicine.

The project was presented to the local ethic committee and was exempted from the need of ethical approval.

The definition of the quality indicators and operational characteristics for civil rescue missions during terror attacks were picked in a way that allows for a quantitative and qualitative description, evaluation and assessment of these missions. The main focus was based on the relevance, the comprehensibility and the measurability of the parameters. These criteria were based on the so called RUMBA norm, which is an acronym for “relevant”, “understandable”, “measurable” “behavioural” (influenced through behaviour) and “achievable”. The process was a mixture of the nominal group technique combined with aspects of the broad Delphi method. Through this approach we could integrate the abundant background knowledge and experience of our experts and combine it with the benefits of group dynamics and discussion.

In the first meeting the 14 dedicated members of the expert group were asked to define quality indicators and operational characteristics to best describe and evaluate rescue missions during mass killing events. In order to obtain a more general approach a direct relation to the Wuerzburg mission should be avoided at that time. The result was a list of indicators and characteristics which were than judged and adopted by consensus within the group. A second meeting took place after two weeks. Each indicator was again critically evaluated within the working group and the final template was approved by full consensus [10]. After the quality indicators were ascertained, they were placed in clusters based on important sub-classifications of the emergency mission in order to allow for a clear and structured arrangement and overview (third meeting). This result was presented to an independent expert for review and validation.

In the presented study, we followed a three-step evaluation process:

1. Systematic data collection according to the quality indicators and operational characteristics
2. Systematic stratification and assessment of the data
3. Mission specific prioritisation and definition of the lessons learned

Primary data of the emergency rescue mission was collected and transferred into an evaluation matrix. A three point rating system was used for the assessment of the results. For each quality indicator we attempted to define a specific rating description (e.g. 0 points = not fulfilled, 1 point = partially fulfilled, 2 points = fully fulfilled).

We undertook a great effort to attribute an individual grading system to each indicator wherever possible. It was impossible to do this in a uniform way as every indicator and the corresponding result had its own meaning. For example: in Table 1 “mission related data”, the first line raises the question at which time it was noticed that it was a life threatening incident for the rescue services. The result was 6 min after the first emergency call. This Δt on its own is not indicative of any consequences. Therefore, we developed the grading system exclusively for this indicator:

- 2: notification prior to arrival of the first operational forces (this means early enough to draw security related consequences).
- 1: notification after the arrival of the first operational forces, no threat for the operational forces (this means to late but without consequences for the rescue forces).
- 0: threat or damage to the operational forces (this means to late with significant and negative consequences for the rescue forces).

**Table 1:** General Characteristics

General Characteristics	Data	Assessment
Date	2016.07.18	descriptive
Weekday and time of day	Monday 09:14 pm	descriptive
Bank holiday	no	descriptive
Weather (rain, snow etc.)	20 °C, cloudy dry	descriptive
Place of incident - rural area or city?	City of Wuerzburg (130.000 inhabitants)	descriptive
Number of hospitals in the area (radius 50 km)	16	descriptive
Number of local trauma centers?	4	descriptive
Number of regional trauma centers?	3	descriptive
Number of national trauma centers?	1	descriptive
*Is there a written concept for Mass Casualty Incidents for the emergency medical services in place?*	*yes*	*See lessons learned*
*Is there a written and coherent concept for dealing with mass killing incidents/life threatening mass casualty incidents*	*yes*	*See lessons learned*
Total number of casualties	8	descriptive
Number of casualties classified as T1/RED in medical triage	4	descriptive
Number of casualties classified as T2/YELLOW in medical triage	1	descriptive
Number of casualties classified as T3/GREEN in medical triage	1	descriptive
Number of casualties classified as T4/BLACK in medical triage	1	descriptive
Number of hospitalised casualties	6	descriptive
Number of casualties deceased on site	1 (the offender)	descriptive
Number of uninjured people who have been affected by the event	15	descriptive

For some results such an assessment was not reasonable and some results were descriptive values and therefore the developed rating system was not applicable. (Tables 1, 2, 3, 4, 5, 6, 7, 8, 9, 10, 11, 12 and 13).

**Table 2:** Mission related data

Mission related data	Data	Assessment
At what time was it noticed that this was a life threatening incident for the rescue services? (Δt from first emergency call to notification of life threatening situation)	09:20 pm Δt: 6 min	Result: 2 2: prior to arrival of the first operational forces 1: after the arrival of the first operational forces, no threat to the operational forces 0: threat or damage to the operational forces
Did first notification of the incident happen through police, rescue headquarter or operational forces on scene?	Police headquarter	descriptive
Δ t from notification until communication between rescue headquarter and police headquarter	Δt: 0 min	Result: 2 2: prior to arrival of the first operational forces 1: after the arrival of the first operational forces, no threat to the operational forces 0: threat or damage to the operational forces
Δ t from alarm until successful notification of all operational forces about life threatening situation	Δt: 0 min	Result: 2 2: prior to arrival of the first operational forces 1: after the arrival of the first operational forces, no threat to the operational forces 0: threat or damage to the operational forces
Δ t from alarm until arrival on scene of the first operational forces	Δt: 7 min	Result: 2 2: within the help period 1: outside the help period due to incident circumstances 0: outside the help period without justification
Δ t from arrival on scene until first understanding of the situation and report to the rescue headquarter	Δt: 1 min	Result: 2 2: immediate information 1: delayed information 0: no information
Δ t from arrival of the first operational forces until the first assessment and tactical decision	unknown	
Δ t from arrival of the mission commander until first assessment of situation and planning of the mission	Δt: 10 min	Result: 2 2: immediate 1: delayed 0: no assessment and planning
Δt from first notification of a life threatening situation until threat cessation	Δt: 106 min	descriptive
Use of guns? Use of thrusting weapons (knife etc.)?	Knife and axe	descriptive
Use of explosive agents i.e. Improvised Explosive Devices?	no	descriptive
Abuse of vehicles (truck/car)?	no	descriptive
Chemical, biological, radio-nuclear threats (CBRN)	no	descriptive
Any other imminent danger?	no	descriptive
Total time of mission (in minutes)	245 min	descriptive
Static situation (no change in circumstances such as mobile offenders, changing threats and growing number of casualties)?	No	descriptive
Dynamic situation (change in circumstances such as mobile offenders, changing threats and growing number of casualties)?	yes	descriptive
Combination of static and dynamic situation?	no	descriptive
Multisite attack?	no	descriptive

**Table 3:** Alarm Procedure

Alarm Procedure	Data	Assessment
Δ t from first emergency call until use of buzzword (i.e. terror, rampage, life-threatening situation)	Δt: 4 min	Result: 2 2: immediate 1: delayed 0: no
Δt from first emergency call until first alarm	Δt: 4 min	Result: 2 2: immediate 1: delayed 0: no
Δ t from first alarm until rescue forces report operational readiness	Δt: 20 min	descriptive
Δ t from operational readiness until arrival of the rescue forces on scene	Δt: 10 min	descriptive
Was there enough manpower at the rescue headquarter (dispatch center) at the start of the operation	No	descriptive
Has the rescue headquarter an alarm system of additional recruitment in place if needed	yes	descriptive
If there was a lack of forces in the rescue headquarter: Δ t from first notification until full recruitment	Δt: 30 min	descriptive

**Table 4:** Organization of the scene (*distance in meters)

Organization of scene (Arrangement of the area)	Data	Assessment
*Was there a structured organization of the scene during the operation?*	Yes	*Result: 1* *1: Yes* *0: No* *See lessons learned*
If so, who coordinated the organization of the scene (police forces and/or rescue forces)?	Rescue Headquarter	descriptive
Δ t from first alarm until arrangement of the area	Δ t: 0 Minutes	Result: 2 2: prior to arrival of the first operational forces 1: after the arrival of the first operational forces, no threat for the operational forces 0: threat or damage to the operational forces
What was the arrangement of the area?	Yes: Damage sector Deployment sector On–site Command post	descriptive
*Was the* arrangement of the area *adequate for life threatening incidents (*e.g. *unsafe and safe zones, safe assembling areas)*	*No, due to the dynamic situation the deployment (EMS) sector was located in the unsafe zone*.	*Result: 0* *2: adequate* *1: not adequate, no threat for the operational forces* *0: potential threat or damage to the operational forces* *See lessons learned*
*Did everyone know (police forces and rescue forces) about the organization of the scene?* *Was the organization of the scene applied successfully?*	*Yes!* *No: due to ambigous information*	*See lessons learned*
Δ t from first alarm until a deployment sector was planned	Δ t: 0 Minutes	
Distance (*in meters) between the deployment sector to the unsafe zone	0 m	Result: 0 1: sufficient 0: insufficient
Distance* between the on-site command post to the unsafe zone	500 m	Result: 1 1: sufficient 0: insufficient
Was a triage area established?	no	
If so - distance* between triage area and unsafe zone? (secured perimeter)	not applicable	
Was there an assembling area (casualty gathering point) established	no	
If so – distance * between assembling area (casualty gathering point) and unsafe zone?	not applicable	
Was there a zone-related stepwise medical treatment concept according to the principals of Tactical Combat Casualty Care (care under fire, tactical field care, tactical evacuation care) established?	No, the full medical care was provided in the unsafe area	descriptive
Was an assembly area for uninjured people, who have been affected by the event, established?	yes	descriptive
If so - distance* between the assembly area and unsafe zone?	2000 m	Result: 0 1: sufficient 0: insufficient

**Table 5:** Mission strategy and tactics

Mission strategy and tactics	Data	Assesment
*Which mission strategy* i.e. *“Clear up the scene”- was applied?*	*No terror related strategy was applied. A mission strategy for mass casualty incidents was applied.*	*See lessons learned*
*Which tactical plan of action was chosen?* *For example:* *Fastest transport to hospitals of the patients with Triage Category T1/RED?*	*Basically the mission strategy for mass casualty incidents was applied. There was an ongoing threat, so swift evacuation of the patients with triage category I/RED was the most important tactical decision*	*See lessons learned*
At what time were tactical consequences drawn?	unknown	
Who drew them?	Subsection Commander of the “subsection damage”	Descriptive
Δ t from arrival of the first rescue services on scene until a tactical plan of action was fully implemented?	Unknown	
Coordinated tactical decisions drawn by police, emergency medical services and fire brigade?	Yes

**Table 6:** Incident Command System/ Line of Command

Incident Command System/Line of Command	Data	Assessment
Was an incident command system established?	Yes	Result: 1 1: yes 0: no
*Did the incident commander in chief (medical) have influence on the course of action during the mission?*	*Partly*	*Result: 1* *2: complete* *1: partly* *0: none (see lessons learned)*
*Did the command processes work adequately?*	*Partly*	*Result: 1* *2: complete* *1: partly* *0: none (see lessons learned)*
*Did the incident commander in chief (medical) always have adequate information for repeated re-evaluation and understaning of the situation (continuous flux of reports)?*	*No*	*Result: 0* *2: always* *1: sometimes* *0: never (see lessons learned)*
Did the incident commander in chief (medical) have influence on the course of action during the mission	Partly	Result: 1 2: complete 1: partly 0: none
*Did the incident commander in chief (medical) have control of the subsection commanders?*	*Partly*	*Result: 1* *2: complete* *1: partly* *0: none (see lessons learned)*
Was a functioning resource management established?	Yes	Descriptive

**Table 7:** Communication

Communication	Data	Assesment
Was there a previously defined communication infrastructure between the rescue headquarter and the police headquarter?	No	Result: 0 1: present 0 not present
Which communication system was used?	Radio and telephone	Descriptive
Was a communication plan established before the mission started?	No	Result: 0 1: yes 0: no
Was the plan applied?	Not applicable	
*Was there a regular and structured re-evaluation of the situation for the incident commander in chief throughout the different mission phases (continuous report flux)*	*no*	*See lessons learned*
If so, where the results recorded in a timely and structured method (mission diary/ map of incident site)?	no	Result: 0 1: yes 0: no
*Was an infrastructure for communication between medical services, police and fire services established?*	*no*	*See lessons learned*
Did police, medical services and fire services communicate regularly and effectively with each other?	Yes: the subsection commanders No: The incident commanders in chief	1 2: yes 1: partly 0: no
If so, which communication infrastructure was used?	Mobile telephone	Descriptive

**Table 8:** Triage

Triage	Data	Assessment
Was a triage algorithm used?	No specific algorithm was used.	Result: 0 2: the trained standard algorithm 1: any arbitrary algorithm, 0: no algorithm
If so, which triage algorithm was used?	Not applicable The standard triage algorithm in Bavaria is mStART. It was not deployed by the rescue forces.	
Δ t from arrival of first rescue services on scene until the start of triage?	Unknown	2: immediate 1:delayed 0: no triage
Who was responsible for the triage?	Emergency medical services	descriptive
Where life saving measures delivered during the first triage cycle?	Yes	descriptive
Δ t from arrival of first rescue services on scene and communication of primary triage result to the rescue headquarter	Δ t: 8 min	descriptive
Were casualties treated according to triage priorities?	Yes	Result: 2 2: yes, completely 1: yes, partly 0: no
Were casualties allocated to hospital according to triage priority?	Yes	Result: 2 2: yes, completely 1: yes, partly 0: no

**Table 9:** Casualty Care

Casualty Care	Data	Assessment
Was medical care based on damage control principals?	No, there was complete resuscitative care provided	descriptive
Was there a stepwise care provision according to the different sectors (unsafe sector: “Care under fire”; semi-safe sector: “Tactical Field Care”; safe sector: “Tactical Evacuation Care”?	No, the medical care was provided in the unsafe area (due to the circumstances explained above)	descriptive
Were there any delays in medical care due to safety issues such as threats to rescuers?	no	descriptive
If so, how long did it take until the last casualty had received medical care?		descriptive
Δ t from first emergency call until transport of the first casualty with triage category T1/RED to hospital	Δ t: 38 min	descriptive
Δ t from first emergency call until transport of the first casualty with triage category T2/YELLOW to hospital	Δ t:41 min	descriptive
Δ t from first emergency call until transport of the first casualty with triage category T3/GREEN to hospital	unknown	descriptive
Δ t from first emergency call until arrival of the first patient with category T1/RED in hospital	Δ t: 48 min	descriptive
Δ t from first emergency call until arrival of the first casualty with category T2/YELLOW in hospital	unknown	descriptive
Δ t from first emergency call until arrival of the first casualty with category T3/GREEN in hospital	Δ t: 77 min	descriptive
Δ t from first emergency call until transport of the last casualty with categoryT1/ RED to hospital	Δ t: 80 min	descriptive
Δ t from first emergency call until transport of the last casualty with category T2/YELLOW to hospital	none	descriptive
Δ t from first emergency call until transport of the last casualty with category T3/GREEN to hospital	none	descriptive
Δ t from first emergency call until arrival of the last casualty with category T1/RED in hospital	Δ t: 79 min	descriptive
Δ t from first emergency call until arrival of the last casualty with category T2/YELLOW in hospital	none	descriptive
Δ t from first emergency call until arrival of the last casualty with category T3/GREEN in hospital	none	descriptive
Δ t from first emergency call until last casualty found	Δ t:36 min	descriptive
Δ t from first emergency call until identification of all casualties	Δ t:106 min	descriptive

**Table 10:** Documentation

Documentation	Data	Assessment
How were the triage results documented?	EMS (Emergency medical service) Protocol	descriptive
How was the casualty registration documented?	Computer based protocol	descriptive
Δ t from first alarm until full identification of casualties and involved parties documented	Δ t: 106 min	
Were injury report cards used?	Yes	Results: 0 2: for all patients 1: some patients 0: No cards were used
Was the documentation complete?	No	Results: 0 1: complete 0: incomplete

**Table 11:** Rescue Forces

Rescue Forces	Data	Assessment
Was there an adequate casualties / operational force ratio at any point during the mission	yes	Result: 1 1: yes 0: no
If so, was this adequate ratio lost again at some point (too many rescue forces)?	Yes, many of the rescue service forces were kept in reserve as a multisite attack was initially anticipated	
Δ t from first emergency call until adequate ratio was reached	Δ t: 26 min	Result: 2 2: fast 1: with delay 0: never
Were staff reservoirs built up?	yes	
Was a structured replacement of the operational forces necessary?	no	descriptive
Were the operational forces in concrete danger at any point?	yes	descriptive
Were members of the operational forces injured during the mission	no	Result: 1 1: no 0: yes

**Table 12:** Hospitals

Hospitals	Data	Assessment
Δ t from first notification/alerting of the hospitals until arrival of the first casualty	Hospital 1: Δ t = 36 min Hospital 2: Δ *t* = 36 min Hospital 1: Δ t = 36 min	descriptive
Do the hospitals have plans in place?	Hospital 1: yes Hospital 2: yes Hospital 1: yes	Result: 2 2: all 1: partly 0: none
Were the emergency plans activated?	Hospital 1: no Hospital 2: no Hospital 1: no	descriptive
Were the emergency plans successfully applied?	not applicable	
When was the rescue headquarter informed about the capacity of the hospitals to receive and treat casualties? Δ t from first notification until capacity information	Hospital 1: 6 min Hospital 2: 5 min Hospital 3: 5 min	Result: 2 2: immediately 1: delayed 0: never
Ratio between announced to actually delivered causalities	Hospital 1: 4/4 Hospital 2: 1/1 Hospital 3: 1/1	descriptive
Adequate allocation and distribution of causalities?	yes	Result: 2 2: yes - all 1: yes - partly 0: none
Number of self-referred casualties	zero	descriptive
Did the hospitals have a strategy in place to deal with life threatening mass casualty incidents?	Hospital 1: no Hospital 2: no Hospital 3: no	Result: 0 1: yes 0: no

**Table 13:** Psychological emergency care

Psychosocial emergency care	Data	Assessment
Δ t from first alarm to deployment of the psychosocial emergency services	Δ t: 7 min	Result: 2 2: early 1: late 0: never
Was the psychosocial emergency care coordinated through police and medical service?	yes	descriptive
Was psychosocial emergency care offered during the days after the incident?	yes	descriptive
Was psychosocial emergency care offered to rescue forces during the days after the incident?	yes	descriptive
Was psychosocial emergency care offered to affected people during the days after the incident?	yes	descriptive
Was there a felt/real hazard to the rescue forces at any point?	yes	descriptive
How many casualties did receive psychosocial support?	none	descriptive
How many uninjured affected people did receive psychosocial support?	20	descriptive
How many members of the rescue forces did receive psychosocial support?	67	descriptive

After the primary data collection and stratification, the actual evaluation of the terror attack and the formulation of the lessons learned started. For prioritisation purposes those parameters, which were seen as particularly relevant to the terror attack in Wuerzburg, were identified in each cluster by the group of experts.

## Results

As a result of this process, 158 identified quality indicators were categorised into 13 clusters [10]

Tables 1, 2, 3, 4, 5, 6, 7, 8, 9, 10, 11, 12 and 13 show the quality indicators and operational characteristics in their allocated clusters. Some of the quality indicators which were very specific to the German Emergency Medical Service system have been omitted in this publication in order to accommodate for a more international audience. At the end, 139 quality indicators remained for the presentation of the evaluation process.

The quality indicators, the data of the Wuerzburg emergency rescue mission and the assessment of the data are shown in Tables 1, 2, 3, 4, 5, 6, 7, 8, 9, 10, 11, 12 and 13.

The quality indicators, which have been identified as most important by the group of experts (by majority opinion) for the Wuerzburg emergency rescue mission formed the basis for the formulation of the lessons learned.

### Category: General Characteristics (Table 1)

- Is there a written concept for mass casualty incidents for the EMS?
- Is there a written and comprehensive concept for dealing with life threatening incidents?

The EMS of Wuerzburg had a regional operational concept for mass casualty incidents. The concept was available in written form and had been taught and practised previously. The concept was principally useful in handling the situation but it was not specifically outlined for life threatening incidents.

The Bavarian Ministry of the Interior and the ministry for Building and Transport had additionally, very recently (June 2016), published a new concept named REBEL to deal with life threatening incidents.

### Problem Identified

The very short availability of REBEL, meant that the concept had not been taught and practiced; hence it could not be used during the terror attack and hence the regional mass casualty incident concept was used instead. Additionally medical equipment for the care of penetrating injury (tourniquet, chest seal etc.) was not available at the time of the assault.

### Lesson learned 1

A harmonised, easy to use and well-trained concept is essential in order to successfully deal with life threatening mass casualty incidents. The REBEL concept has existed for over a year now (2016–2017) and the ambulances have been equipped with additional material according to the concept in order to provide adequate prehospital “damage control” care (tourniquets, chest seals, pelvic belts, haemostatic bandages, IO needles and emergency thoracostomy needles). The concept was adapted and modified to fit the specific regional requirements around Wuerzburg and the training phase has been completed successfully.

### Category: Mission related data (Table 2 )

- When was it noticed that this was a life threatening situation for the operational forces? (Δ t from first alarm to notification)
- Δ t from notification until communication between medical rescue control centre and police control centre
- Δ t from alarm until successful notification of all operational forces about life threatening situation

The first communication between the police control centre and the medical rescue control centre included the word “rampage” and “terror” and hence the situation was clearly defined from the start. The communication between police and emergency medical service (EMS) worked very well in the initial phase of the mission. All relevant information was passed on between the different services.

### Problem identified

During the course of the mission a one sided information flow between the police control centre and the rescue control centre was established. Unfortunately the communication was uncoordinated and led to a diffusion of information as many phone calls involving many different parties were held. Information could not be gathered effectively which led to a lack of concise information, evaluation and situational understanding. The drawing of concise consequences and conclusions were impossible.

### Lesson learned 2

Establishment of a high priority communication infrastructure between the police control centre and the medical rescue control centre (“red phone”) is a crucial point. This is vital in order to provide a constant flow of information between a defined dispatcher and recipient. This communication is essential not only during the initial phase (early and fast track evaluation of the situation via this communication channel) but throughout the mission. The aim is to provide continuity of information between few well defined individuals in order to allow information gathering instead of information dispersal.

### Category: Organization of the scene (arrangement of the area) (Table 4)

- Was there a structured organization of the scene during the operation?
- Was the organization of the scene adequate for life threatening incidents (e.g. unsafe and safe sectors, safe assembling areas?)
- Did everyone know (police forces and operational medical forces) about the organization of the scene?
  Was the organization of the scene applied successfully?

Yes – there was a structured organization of the scene during the operation.

### Problem identified

Because of the mobile and dynamic incident scene (running train) and the ambiguous description of the defined deployment sector, the deployment sector of the EMS was placed within the unsafe area despite the police having designated a deployment sector within the safe zone. This problem was based on miscommunication and misunderstanding between the police and the EMS rather than difficulties with the organization of the scene itselfs. As a result casualties were treated by the EMS within the unsafe, dangerous zone of the incident with a still uncontrolled, active perpetrator. Fortunately none of the rescue forces got injured and the treatment of the casualties could take place without any delay.

### Lesson learned 3

A commonly (police and EMS) decided and consented organization of the scene and arrangement of the area was identified as a crucial point for the successful completion of the mission.

- Establishment of a common access route to the incident area used by police, firearm services and emergency medical services.
- Establishment of a common arrangement of the area especially a common and safe deployment sector.
- Establishment  of a common on-site command post.
- The incident area should be divided into an unsafe, semi - safe and safe zone with different modes of action within these zones.

### Category: Mission strategy and tactics (Table 5)

- Which strategy i.e. “Clear up the scene”- was applied?
- Which tactical plan of action was chosen?

No strategy specifically designed for life threatening incidents was used. To clear up the scene immediately means to rapidly identify the most seriously injured patients and evacuate them towards the designated hospitals. This strategy reduces the on-scene time and augments the safety of the rescue forces. No such strategy was developed and trained prior to the event and hence none could be applied.

The medical care was based on individual needs and was based on individual injuries rather than tactical considerations.

### Problem identified

Even though it was known that the incident was a terror attack the principles of Tactical Combat Medical Care (TCCC) were not used. These principles were neither well known nor practiced prior to the incident and hence not part of the routine action pattern of the rescue forces. Hence, none of these principles were used during the Wuerzburg terror attack. In terms of safety issues for the rescue forces this would have been beneficial, looking at the quality of medical care however, there was no disadvantage for the casualties.

### Lesson learned 4

In order to allow the application of specific strategies and tactics in a life threatening mission, these strategies and tactics need to be developed and practiced beforehand. Without instructions, knowledge and specific training none of the strategies can be used on scene in a life threatening situation. The operational forces need to have internalised the processes in order to access them effectively in a high pressure situation such as a life threatening mission.

We have formulated the following key points to help preparing the rescue forces for future incidents:

- Are there comprehensive management plans in place?
- Are these plans well known and well trained?
- Are there common drill and training possibilities?
- Are there clear mission goals and strategies?
- Is there stockpiling of the equipment and material?
- Is this equipment and material readily available?

### Category: Incident command system and communication (Tables 6, 7)

Because of the interconnections between these clusters and different quality indicators it has been decided to illustrate them in combination instead of addressing them individually.

- Did the command processes work adequately?
- Did the incident commander in chief (medical) always have adequate information for repeated reevaluation and understanding of the situation (continuous flux of reports)?
- Did the incident commander in chief (medical) have control of the subsection commanders?
- Was there a regular and structured re-evaluation of the situation for the incident commander in chief throughout the different mission phases?
- Was an infrastructure for communication between medical services, police and fire services established?

Functioning command processes could only partially be established. There were functioning command processes between the commanders of the mission subsectors but the on-site incident commander in chief did not have adequate information in order to repeatedly re-evaluate the situation. There was no continuous flux of reports from the subsector commanders (sector danger and sector mission) to the on-site incident commander in chief. There was no infrastructure for communication between EMS and the police.

### Problem Identified

There was an infrastructure for communication between the EMS while this infrastructure was missing for the communication between police forces and EMS.

There was effective communication between the subsector commanders while the communication between the incident commander in chief and the subsection commanders was incomplete.

### Lesson learned 5

A successful mission tactic requires continuous flux of reports to the on-site command post. This includes “up to date” situational information from all different sub sectors of the mission as well as reliable information about the safety situation at the incident site. A predefined and common communication infrastructure for all operational forces is a crucial point for the successful completion of the mission. Use of the same nomenclature between police services and the EMS is a crucial point.

Hospitals play a vital role in the treatment of patients in the case of terrorist attacks. They are a key element in order to evacuate patients from an unsafe environment towards definitive medical treatment. Therefore hospitals need to be involved in the medical response from the very beginning.

## Discussion

This work describes the systematic evaluation of and formulates the lessons learned from a civil rescue mission during a terror attack in Germany.

Applying the described three step evaluation process allowed for an objective, systematic and well-structured description of the rescue mission. The combination of expert input into prioritisation of various quality indicators and the definition of the lessons learned based on those indicators allowed for a very precise and effective evaluation of the Wuerzburg emergency rescue mission.

On the basis of the template of Fattah et al. [8] the rescue mission during the terror attack in Utoja, Norway was evaluated and reported systematically (http://majorincidentreporting.net/wp-content/uploads/2016/08/utoya.pdf). The key lessons are described as follows:

- Lack of a common triage system
- Delayed access to patients due to security issues
- Communication breakdown

Solutions for these lessons learned were:

- The establishment of a national triage system.
- A better system for cooperating with the police forces in order to get quicker access to casualties.
- The improvement of the communication systems.

The close cooperation between police forces and rescue forces in particular, is a key lesson we also defined from our results. Our findings also confirm the need for a secured and coordinated communication.

Two recent publications report specific indicators as quality control tools for major incidents. Nilsson et al. defined a set of 11 performance indicators qualifying the performance of the initial medical command and control in major incidents [11]. Radestad et al. presented key indicators for disaster medical response defined through a Delphi study [12]. Both classification systems are not specific for terror attacks. There is a recent systematic literature review for prehospital management of mass casualty civilian shootings published by Turner et al. in 2016 [13]. None of the included reports was based on a systematic evaluation approach using quality or performance indicators. The authors of the systematic review stated that a uniform and comprehensive reporting of such events is required [13].

Fattah et al. published a systematic review of the literature in 2013 [7]. The authors studied the availability of templates suitable for the report of prehospital major incidents. They identified 10 templates published in the included literature. DISAST-CIR was one of the described templates in this review, which is a standardized template used in Israel and was published by Leiba et al. [9]. Fatah et al. concluded in their systematic review that some limitations are present in all of the templates but that the identified templates may serve as a basis for designing a template that exclusively targets prehospital care in major incidents [7]. Such a template was designed and published by Fatah et al. in 2014 [8]. The authors defined 48 different variables (with 293 sub-variables) in 6 clusters in order to document and describe rescue missions during mass casualty incidents [8]. After the introduction and the subsequent application of the template to several incidents, some limitations were noted by Fattah et al. [14]. Consequently, the authors revised the template, which is now available less detailed and more user-friendly [6]. As we explained above we designed a new template for the evaluation of the Wuerzburg terror attack in order to particularly concentrate on the terror related aspects of the attack. Nevertheless we fully support the goals of Fatah et al. and other authors [5, 6, 13] to significantly increase and improve the disposition to systematically report and evaluate major incidents but also mass killing events. In the future a close international collaboration between the different researchers and caregivers dealing with the issue might help to find the best template in order to improve these missions outcome.

The evaluation process of the Wuerzburg terror attack and the formulated lessons learned are seen as part of a national and international process to improve the management of life threatening mass casualty incidents.

The German Society of Anaesthetics and Intensive Care Medicine (DGAI) has, in collaboration with leading representatives from police, fire brigades, emergency physicians, emergency medical services, hospital services and politics identified systemic issues related to missions during life threatening mass incidents. They have furthermore developed concrete solutions [15].

The German Society of Trauma and Orthopaedics have developed a course concept (Terror and Disaster Surgical Care) with the help of the military, which is now offered and trained on a national level in order to implement the principles of Tactical Abbreviated Surgical Care [16].

There are multiple international publications about the terror attacks in France and their consequences. One specific publication addresses a few points which were deemed particularly important [3]. These points were:

- Management of Uncertainties
- Management of Victims
- Management of Teams
- Communication

Especially the communication topic was very important in the definition of our lessons learned. So we concluded that a predefined and common communication infrastructure (including redundancy) for all operational forces is a crucial point for successful completion of the mission.

Carli et al. have recently published the most important lessons from the most recent terror attacks in France [4]. This publication did not only identify the weaknesses and problems but also described their solutions and current implementation stages of these suggested solutions.

In this, the French group of experts have proven great advancement and their results can be seen as role model for other countries having to deal with terror.

The most important weaknesses and problems are summarised below:

- The handling of war weapon injuries
- The implementation of the preclinical concept
- The handling of trauma in children
- The reaction to chemical weapons
- The assault on hospitals
- Medical treatment “Care under fire”
- Triage in hospital
- Terror attacks in rural areas
- The casualty identification approach
- Psychosocial emergency care provision

Carli states important points which have not played a major role in our evaluation process. This mainly concerns a high number of injured children, attack with chemical weapons and the casualty identification process. The integration of the lessons learned into the education of medical students is also a very important task mentioned by Carli et al. [4].

### Limitations

No standardised Delphi method was used in the ascertainment of the quality indicators. The standardised Delphi method was rejected as the most important characteristic of it is the anonymity of the experts [10]. This could and should not have been preserved in this study as their participation during the event was one of the main criteria why the experts were chosen for the expert panel in the first place.

The results of our analysis include specific elements which are based on the regional characteristics of the German/ Bavarian emergency medical service (EMS). It is important to mention that both the police as well as the ambulance services in Germany are under the direct control of the states. They therefore differ between the 16 different German states, which makes the implementation of a national concept extremely difficult and reduces the comparability to other European countries. The quality indicators however are so generally formulated, that - in our opinion - they could serve as a template for evaluation of life threatening mass casualty incidents in any other international EMS system after country specific adaptation.

The event that motivated the formulation of the quality indicators and the following evaluation process was of limited scale and the number of casualties during the rescue mission was low, however we are convinced that the experiences and the resulting analysis according to quality indicators can be easily applied to mass killing incidents with a much higher number of casualties.

Furthermore the presented evaluation does not include biological, chemical or radio-nuclear threats. The response and the preparation for such events needs further detailed evaluation and planning.

Based on our data and according to the continuous evaluation process of the last years [4, 5, 8, 15–19] we have formulated the following set of measures for the management of mass killing incidents (without respect to biological, chemical or radio-nuclear threats).

### “Response bundle”

- High priority communication between the police control center and the rescue control center via defined communication channels for the best understanding of the situation.
- Immediate definition of a coordinated and harmonised course of action by police forces and all other rescue services.
- For the first responders: Give immediate report to the control centre
- Arrangement of the area and management of the scene – definition and communication of the unsafe zone, definition and communication of casualty collection points, triage area (semi-safe zone) and safe treatment area (safe zone)
- Establishment of command and control structures
- Secure Communication
- Early Involvement of hospitals

### Police Forces:

- Immediate threat diffusion
- Immediate estimation and communication of expected casualty numbers to the rescue forces
- Start casualty treatment and evacuation from the unsafe zone to the semi-safe and safe zones - act as evacuation flow accelerator

### Rescue forces:

- Establishment of triage and treatment area in the semi-safe zone
- Fast triage
- Immediate treatment of potentially survivable life-threatening injuries (exsanguination, airway obstruction, tension pneumothorax) during initial resuscitation
- Identification of casualties with life-threatening non-controllable bleeding
- Prioritised and immediate transport of those “Priority 1” patients to nearby hospitals

### “Preparedness bundle”

- Establishment of common (police and rescue forces) sense about mission goals
- Usage of a common language
- Common drill and training
- Predefinition of secured communication channels
- Stockpiling of medical equipment to treat penetrating bleeding injuries
- Stockpiling of transport equipment to maintain evacuation flow
- Secure communication technologies
- Prepare for uncommon threats (e.g. biological and chemical weapons)
- Prepare for children in mass casualty incidents

A major issue for preparation to mass killing incidents is training and education. The most useful approach would be a multidisciplinary curriculum. Based on our results we would recommend that such a curriculum should include these basic points:

- Development of a common sense (police and rescue forces) about mission goals
- Definition of mission goals and strategy
- Managing the scene
- Command and Control structures
- Communication training between different disciplines
- Basic knowledge and understanding of different mission tactics
- Basic skills-training for the treatment of life threatening bleeding injuries
- Managing the evacuation flow

## Conclusion

This work describes the systematic evaluation of the rescue mission during a terror attack according to defined quality indicators. The aim of this approach is to allow for a coherent and homogenous presentation and evaluation of rescue missions during terror attacks. As the template is currently applied for the evaluation and report of another mass killing incident in Germany it might also be helpful to compare different missions with each other. In the future a close international collaboration might help to find the best database to report and evaluate major incidents but also mass killing events. In times where terror attacks and mass casualty incidents have become more common we should all work together to support the goal – to significantly increase and improve the available possibilities to systematically report and evaluate mass killing events and to learn from the results in order to improve these missions outcome.

## References

[1] Hirsch M, Carli P, Nizard R, Riou B, Baroudijan B, Baubet T (2015). On behalf of the health professionals of assistance Publique_Hôpitaux de Paris (APHP.) the medical response to multisite terrorist attacks in Paris. Lancet.

[2] Haug CJ (2015). Report from Paris. N Engl J Med.

[3] Philippe JM, Brahic O, Carli P, Tourtier J-P, Riou B, Vallet B (2016). French Ministry of Health's response to Paris attacks of 13 November 2015. Crit Care.

[4] Carli P, Pons F, Levraut J, Millet B, Tourtier JP, Ludes B (2017). The French emergency medical services after the Paris and Nice terrorist attacks: what have we learned?. Lancet.

[5] Goralnick E, Van Trimpont F, Carli P (2017). Preparing the next terrorism attack: lessons from Paris, Brussels and Boston. JAMA Surg.

[6] Hardy SEJ, Fattah S (2017). Trials and tribulations: how we established a major incident database. Scand J Trauma Resusc Emerg Med.

[7] Fattah Sabina, Rehn Marius, Reierth Eirik, Wisborg Torben (2013). Systematic literature review of templates for reporting prehospital major incident medical management. BMJ Open.

[8] Fattah S, Rehn M, Lockey D, Thompson J, Lossius HM, Wisborg T. A consensus based template for reporting of pre hospital major incident medical management. Scand J Trauma *Resusc Emerg Med*. 2014;22:5–11. 10.1186/1757-7241-22-5 PMC3922248 24517242

[9] Leiba A, Schwartz D, Eran T, Bluumenfeld A, Laor D, Goldberg A, et al. Disast-Cir: disastrous incidents, interactions and results: application t al large-scale train accident. J Emerg Med. 2009;37:46–50. 10.1016/j.jemermed.2007.09.025 18024063

[10] Wurmb T, Justice P, Dietz S, Schua R, Jarausch T, Kinstle U (2017). Quality indicators for rescue operations in terrorist attacks or other threats: a pilot study after the Wuerzburg terrorist attack of July 2016. Anaesthesist.

[11] Nilsson H, Vikström T, Jonson CO (2012). Performance indicators for initial regional medical response to major incidents: a possible quality control tool. Scand J Trauma Resusc and Emerg Med..

[12] Radestad M, Jirwe M, Castren L, Gryth D, Rüter A (2013). Essential key indicators for disaster medical response suggested to be included in a national uniform protocol for documentation of major incidents: a Delphi study. Scand J Trauma Resusc and Emerg Med.

[13] Turner CDA, Lockey DJ, Rehn M (2016). Pre-hospital management of mass casualty civilian shootings: a systematic literature review. Crit Care.

[14] Fattah S, Agledahl KM, Rehn M, Wisborg T (2016). Experience with a novel, global, open-access template for major incidents: qualitative feasibility study. Disaster Med Public Health Prep.

[15] Hossfeld B, Adams HA, Bohnen R, Friedrich K, Friemert B, Gräsner JT, Gromer S (2017). Zusammenarbeit von Rettungskräften und Sicherheitsbehörden bei bedrohlichen Lagen. Anästh Intensivmed.

[16] Friemert B, Franke A, Bieler D, Achatz A, Hinck D, Engelhardt M (2017). Treatment strategies for mass casualty incidents and terrorist attacks in trauma and vascular surgery: presentation of a treatment concept. Chirurg.

[17] Wurmb T, Kowalzik B, Rebuck J, Franke A, Cwojdzinski D, Bernstein N et al. The prehospital management of mass killing events. Results of a nationwide evaluation process conducted by the Federal Office of Civil Protection and Disaster Assistance. Notfall Rettungsmed 2018. 10.1007/s10049-018-0516-6.

[18] Jacobs LM, Wade DS, McSwain A, Butler FK, Fabbri WE, Eastman AL (2013). The Hartford consensus: THREAT, a medical disaster preparedness concept. J Am Coll Surg.

[19] Autrey AW, Hick JL, Bramer K, Berndt J, Bundt J (2014). 3 Echo: concept of operations for early care and evacuation of victims of mass violence. Prehosp Disaster Med.

